# Single‐Cell Dissection of Inflammatory Suppression and Stemness Plasticity Reveals STMN1‐Associated Malignant Epithelial Cells in Severe Lung Cancer

**DOI:** 10.1111/cbdd.70420

**Published:** 2026-10-21

**Authors:** Yanmei Chen, Sisi Chen, Biao Cheng, Yuzhu Tang, Yiyan Huang, Miao Chen

**Affiliations:** ^1^ Department of Endocrinology The Affiliated Yongchuan Hospital of Chongqing Medical University Chongqing China; ^2^ Chongqing Medical and Pharmaceutical College Chongqing China; ^3^ Department of Oncology Xinqiao Hospital, Army Medical University (Third Military Medical University) Chongqing China; ^4^ Sir Run Run Shaw Hospital, Zhejiang University School of Medicine Hangzhou China; ^5^ Institute of Health Sciences China Medical University Shenyang Liaoning China

**Keywords:** advanced lung cancer with malignant serous effusions (ALC‐MSE), cell‐to‐cell communication, inflammatory signaling, single‐cell transcriptomics, STMN1, tumor stemness

## Abstract

The disease course in patients with advanced lung cancer with malignant serous effusions (ALC‐MSE) is complex, and treatment outcomes are difficult to predict; systematic studies on the tumor stem cell‐like characteristics and regulatory mechanisms of this condition are still lacking. Based on single‐cell transcriptomic data and the TCGA‐LUAD cohort, this study systematically analyzed the functional characteristics and key regulatory networks of malignant epithelial cells in severe lung cancer. Key findings include: epithelial cells in severe lung cancer exhibit significantly attenuated inflammation‐ and immunity‐related signaling pathways (including NF‐κB, TNF, and JAK–STAT signaling pathways), enhanced stem cell‐like properties, and metabolic reprogramming; we identified a highly stem‐like malignant epithelial cell subpopulation, represented by Cluster 3, which is enriched in stem‐like pathways such as Notch, Hippo, TGF‐β, and FoxO, and is accompanied by enhanced mitochondrial metabolism and stress adaptability; STMN1 is a key molecule regulating this stem‐like state, showing a significant positive correlation with stemness scores and mRNAi; cells with high STMN1 expression gain a survival advantage and may maintain their stemness by attenuating CAF/ECM signaling with other cells (COL1A1‐CD44), inhibiting complement activation and antigen presentation signaling (such as C3, ANXA1, and MHC‐II‐related molecules), and enhancing stemness niche signaling (MIF‐CD74/CD44). Furthermore, NELFE, MYBL2, FOXN3, POLR2A, and RAD21 were identified as potential upstream regulators of STMN1. In vitro experiments confirmed that, following induction of stem cell‐like properties, STMN1 was significantly overexpressed in lung adenocarcinoma cells, accompanied by upregulation of related stem cell markers (SOX2, NANOG, OCT4, and CD44). In summary, this study reveals a significantly enhanced stemness state in malignant epithelial cells in advanced lung cancer, accompanied by suppressed inflammatory signaling and enhanced metabolic adaptation, and identifies STMN1 as a key molecule driving stemness maintenance and disease progression.

## Graphical abstract

Using single‐cell transcriptomics of lung adenocarcinoma samples (from normal lung to advanced lung cancer with malignant serous effusions, ALC‐MSE) integrated with the TCGA‐LUAD cohort, this study systematically characterized the functional evolution of malignant epithelial cells across disease stages. As disease progressed to ALC‐MSE, inflammatory and immune signaling pathways (NF‐κB, TNF, JAK–STAT, IL‐17, TGF‐β) were progressively suppressed in epithelial cells, while stemness scores and proliferative programs were significantly elevated. Subclustering and copy number variation analysis identified six malignant epithelial subpopulations; among these, Cluster 3 exhibited the highest stemness (CytoTRACE) score and was enriched in Notch, Hippo, TGF‐β, FoxO, and mitochondrial metabolic pathways. Pseudotime trajectory analysis revealed a stage‐specific transition from an inflammation‐active state (Cluster 0) toward a highly stem‐like, undifferentiated state (Cluster 3/State 4) during disease progression. Multi‐dimensional screening—integrating State 4 DEGs, CytoTRACE correlation, and TCGA‐LUAD mRNAsi association—identified STMN1 as the core stemness regulator, showing highest expression in Cluster 3 and State 4. Cell–cell communication analysis demonstrated that STMN1^+^ malignant epithelial cells attenuate CAF/ECM‐dependent signaling (COL1A1‐CD44, FN1‐ITGAV/ITGB1) and immune recognition pathways (C3, ANXA1, MHC‐II), while enhancing stemness niche signaling (MIF‐CD74/CD44). SCENIC analysis identified NELFE, MYBL2, FOXN3, POLR2A, and RAD21 as potential upstream transcriptional regulators of STMN1. In vitro tumorsphere induction in A549 cells confirmed significant upregulation of STMN1 alongside canonical stemness markers (SOX2, NANOG, OCT4, CD44). These findings reveal that ALC‐MSE is characterized by a coordinated suppression of inflammatory signaling and enhancement of metabolic adaptation, with STMN1 serving as a key node linking stemness maintenance, microenvironmental remodeling, and immune evasion in severe lung cancer.

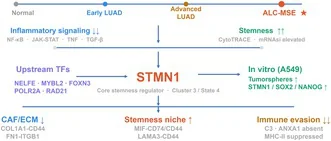

## 1 Introduction

Lung cancer is the leading cause of cancer‐related deaths worldwide, and lung adenocarcinoma (LUAD) is the most common histological subtype, accounting for approximately 40%–50% of all lung cancers (Sung et al. 2021; Zhang, Dai, et al. 2022). Among patients with advanced LUAD, some rapidly progress to a critical condition characterized by severe impairment of multiple organ functions, including respiratory and circulatory systems, with an extremely poor clinical prognosis (Meert et al. 2017; Soares et al. 2014). To better identify and manage these high‐risk patients, the new clinical concept of “Severe Lung Cancer” has emerged. According to the 2026 “International Consensus on Severe Lung Cancer (Second Edition),” this concept primarily refers to patients with an Eastern Cooperative Oncology Group (ECOG) Performance Status (PS) score of 2–4. In these patients, tumor‐related complications, treatment‐related adverse events, and acute comorbidities often intertwine, resulting in a complex clinical course, difficulty in predicting treatment response, and extremely short survival (Lin et al. 2026). However, current understanding of the molecular pathophysiology of severe lung cancer remains very limited. Therefore, elucidating its driving mechanisms at the molecular level and identifying key biological characteristics are of significant clinical importance for improving precision diagnosis and treatment strategies for these patients.

Cancer stemness refers to the ability of tumor cells to acquire stem cell‐like properties, including the potential for self‐renewal and multipotent differentiation. Cancer stem cells (CSCs) with high cancer stemness are the primary source of tumor heterogeneity and play a central role in treatment resistance, metastasis, and recurrence (Dakal et al. 2024; Loh and Ma 2024). Tumors in advanced stages often exhibit more aggressive phenotypes, such as low differentiation, high proliferation, and treatment resistance; these characteristics are highly consistent with tumor stemness (Shang et al. 2026). Recent studies have shown that stemness is not a fixed characteristic, but rather a highly plastic cellular state; under microenvironmental stresses such as hypoxia, chemotherapy, and oxidative stress, differentiated tumor cells can undergo dedifferentiation and regain a stem cell‐like state (Dirkse et al. 2019). Signaling pathways such as Notch, Wnt/β‐catenin, PI3K/AKT/mTOR, Hippo, TGF‐β, and FoxO work together to maintain the stem cell‐like characteristics of CSCs by regulating their self‐renewal, multipotent differentiation, and anti‐apoptotic capacity (Chu et al. 2024). Patients with advanced lung cancer often experience systemic hypoxia, acidosis, a cytokine storm, and microenvironmental remodeling induced by multi‐line therapy, all of which contribute to an extremely adverse microenvironmental stress (Ponholzer et al. 2025; Sackstein et al. 2021). Studies have shown that these extreme microenvironmental stresses can induce enhanced stemness in CSCs via the HIFs and NF‐κB signaling pathways: acidosis (pH 6.8) enhances CSC self‐renewal and mitochondrial function through the CYP24A1 and G6PD/H6PD pathways (Hu et al. 2019; Xu et al. 2021). Intermittent hypoxia upregulates Oct4, Sox2, and Nanog via the HIF/NF‐κB pathway and reshapes the immune microenvironment (Zhang et al. 2026); NF‐κB, which is located upstream of HIFs in the hypoxia network, serves as a central hub linking hypoxia to CSCs (Kannappan et al. 2022). The complex microenvironment resulting from these factors, including persistent hypoxia, chronic inflammation, and immune dysfunction, creates a “stem‐like” niche conducive to tumor cell survival, driving lung cancer toward an extreme disease state and even into a critical condition that is difficult to treat.

Although inflammation and hypoxia can participate in the maintenance and regulation of CSC fate by providing appropriate signals within the microenvironment (Cabarcas et al. 2011). However, there is currently a lack of systematic studies at single‐cell resolution to determine whether the enhanced stemness of tumor cells in severe lung cancer is driven by specific microenvironmental factors such as inflammation and hypoxia. It also remains unclear whether the stemness regulatory mechanisms observed in ordinary lung cancer can be extrapolated to severe disease states. Therefore, elucidating the alterations in inflammatory signaling, stemness characteristics, and microenvironmental regulatory networks of malignant epithelial cells in severe lung cancer is of great significance for gaining a deeper understanding of disease progression mechanisms and identifying new therapeutic targets.

Based on this, this study utilizes single‐cell transcriptomics in conjunction with the TCGA‐LUAD transcriptomic cohort to systematically analyze the stemness characteristics and potential regulatory mechanisms of malignant epithelial cells in advanced lung cancer at the single‐cell level. The objectives are: (1) to identify a high‐stemness subpopulation of malignant epithelial cells in advanced lung cancer and further investigate whether alterations in immune and inflammatory signaling are present; (2) to screen for key molecules involved in maintaining the stemness of high‐stemness malignant epithelial cell subpopulations; (3) to investigate the potential role of STMN1 in the regulation of stemness in lung adenocarcinoma and its upstream and downstream regulatory mechanisms. This study is expected to provide a new theoretical basis for understanding the molecular mechanisms of advanced lung cancer and to serve as a reference for the development of relevant precision treatment strategies.

## 2 Materials and Methods

### 2.1 Data Collection and Download

The single‐cell data for lung adenocarcinoma were obtained from dataset GSE131907 in the GEO database (https://www.ncbi.nlm.nih.gov/geo/browse/). This dataset contains single‐cell sequencing data from 58 lung adenocarcinoma samples collected from 44 patients, including samples from normal lung tissue (nLung), early‐stage lung adenocarcinoma (tLung), advanced lung adenocarcinoma (ALC, tL/B). Additionally, advanced lung cancer with malignant serous effusions (ALC‐MSE, tL/B_MSE) samples were identified from the advanced lung cancer samples based on the identifier EBUS_06.

LUAD transcriptomic data was downloaded from the TCGA database using TCGAbiolinks (v2.32.0), and data acquisition and preprocessing were completed using the GDCdownload and GDCprepare functions. FPKM expression levels were extracted and normalized using log2(FPKM+1). Version numbers were removed from Ensembl gene IDs, which were then converted to Gene Symbols based on the Ensembl database. Genes that were not successfully annotated or were duplicates were excluded, resulting in a standardized expression matrix. The dataset initially contained 601 samples; after screening, 540 tumor samples were retained for subsequent analysis.

### 2.2 Dimension Reduction, Clustering, and Cell Annotation of Single‐Cell Sequencing Data

Dimension reduction and clustering analysis of single‐cell data were performed using the Seurat package (ver. 5.4.0). First, the data were normalized using the “NormalizeData” function, and 2000 highly variable genes were identified. Next, the “decontX” function was used to assess the level of contamination from environmental free RNA, and low‐quality cells with a contamination rate > 0.2 were removed. The Quality Control (QC) criteria were as follows: mitochondrial gene proportion ≤ 20%, UMI counts between 100 and 150,000, and detected gene counts between 200 and 10,000. Batch effects were corrected using “RunHarmony,” and the data were scaled using “ScaleData,” with mitochondrial gene proportion and cell‐cycle scores (S.Score and G2M.Score) regressed out. Cell‐cycle regression was applied only to the scaled data used for dimensionality reduction and clustering and did not affect the normalized RNA expression matrix used for differential expression analysis.

Using the 2000 highly variable genes as input, principal component analysis (PCA) was performed using the “RunPCA” function. The top 15 principal components were selected to construct a shared nearest neighbor (SNN) network, and clustering analysis was conducted using the “FindClusters” function. Cluster stability was evaluated at different resolutions using “clustree” (v0.5.1), and a clustering resolution of 0.7 was ultimately determined. Finally, the clustering results were visualized using the “RunUMAP” function. Cell annotations for each cluster were performed by referencing classic marker genes for different cell types obtained from the CellMarker platform (http://bio‐bigdata.hrbmu.edu.cn/CellMarker/) and incorporating findings from previous studies (Zhang et al. 2024).

### 2.3 Independent Validation

An independent LUAD single‐cell RNA‐sequencing cohort (GSE189357) was used to validate the association between STMN1 and malignant epithelial cell stemness. Cells with 300 < nFeature_RNA < 7500 and mitochondrial transcript proportion < 25% were retained, and cells with a decontX contamination fraction > 0.2 were excluded. After Harmony batch correction, cells were clustered and annotated using canonical markers. Epithelial cells were subsequently reclustered, and inferCNV was performed using endothelial, T/NK, and B cells as non‐malignant reference populations to identify putative malignant epithelial cells. CytoTRACE2 was then applied to assess their relative differentiation and stemness states, followed by evaluation of STMN1 expression across malignant epithelial subclusters.

### 2.4 Differential and Enrichment Analysis

Differential expression analysis was performed using Seurat FindMarkers with the default Wilcoxon rank‐sum test (min.pct = 0.01, logfc.threshold = 0.1). Genes with adjusted *p* < 0.05 were considered differentially expressed, and avg_log2FC was used to represent the direction and effect size of expression changes. Patient‐ and batch‐level variables were not included as additional covariates. For GSEA, FindMarkers was rerun with logfc.threshold = 0, and genes were ranked by avg_log2FC. GSEA and KEGG enrichment analyzes were performed using clusterProfiler (v4.12.6) with human KEGG pathways retrieved online. GSEA used the adaptive fgseaMultilevel algorithm without a fixed number of permutations, with gene set sizes restricted to 10–500 genes and FDR < 0.05 considered significant.

### 2.5 Epithelial Cell Subclustering and CNV Analysis

After epithelial cells were extracted, 2000 highly variable genes were selected for subclustering using Seurat. The data were scaled with ScaleData, and the first 15 principal components were used for clustering at a resolution of 0.1. Large‐scale CNVs were inferred using fastCNV, with epithelial cells from normal lung samples used as the reference. The fastCNV‐derived cnv_fraction was used to quantify CNV burden, and the mean, median, and proportion of cells with cnv_fraction > 0.3 were calculated for each cluster. Malignant epithelial populations were annotated by integrating CNV burden, tumor‐sample origin, and marker expression, including epithelial markers (EPCAM, KRT18, and KRT19), normal lung epithelial markers (AGER, SFTPC, and LAMP3), and tumor‐associated markers (CEACAM5, CEACAM6, and MUC1) (Kim et al. 2022, 2020; Li et al. 2022).

### 2.6 Stemness Scoring and Functional Analysis of Malignant Epithelial Cell Subpopulations

The study used CytoTRACE (v1.1.0) to analyze the stemness of malignant epithelial cells. CytoTRACE is an unsupervised algorithm based on single‐cell transcriptomic data that predicts relative differentiation status; it assigns each cell a stemness score ranging from 0 to 1, where a higher score indicates greater stemness and lower differentiation, and vice versa. CytoTRACE scores exhibit significant heterogeneity among tumor cells (Zhang, Wang, et al. 2022; Zhao et al. 2025). In addition, the FindAllMarker function was used to identify genes specifically expressed in malignant epithelial cell subpopulations for KEGG functional enrichment analysis, with the aim of exploring the functions of these subpopulations.

For the TCGA‐LUAD cohort, we used Synapser (v2.1.5.356) to download stem cell expression profile data (syn2701943) from the Precursor Cell Biology Consortium (PCBC) database and constructed a stemness feature model based on the One‐Class Logistic Regression (OCLR) algorithm. Subsequently, using the stemness feature gene weights obtained from training, the mRNA expression‐based stemness index (mRNAsi) was calculated for TCGA‐LUAD tumor samples. Spearman's correlation was used to assess the similarity between sample expression profiles and stemness features, and the mRNAsi was normalized to the range of 0–1 using the Min‐Max method. Higher mRNAsi values indicate stronger stemness characteristics in tumor cells.

To assess the potential confounding effect of cell proliferation on the association between STMN1 and stemness, Spearman correlations were calculated between STMN1 expression and MKI67, TOP2A, S.Score, and G2M.Score. Partial correlation analysis between STMN1 expression and CytoTRACE2 scores was further performed using the ppcor package (v1.1), adjusting for S.Score and G2M.Score. Univariate linear regression models were used to compare the variance in CytoTRACE2 scores explained by STMN1, MKI67, and TOP2A expression. In addition, malignant epithelial cells were stratified into low‐ and high‐proliferation groups based on, and the STMN1–CytoTRACE2 correlation was assessed separately in each group.

### 2.7 Pseudo‐Time Series Analysis

After identifying malignant epithelial cells and completing the stemness scoring, the Seurat objects representing the malignant epithelial cells are converted to the CellDataSet format required by Monocle2. Subsequently, the data is normalized and dispersion is estimated using the “estimateSizeFactors()” and “estimateDispersions()” functions. High‐variability genes with an expression mean ≥ 0.01 and an observed dispersion no lower than the fitted dispersion are then selected for trajectory ordering. Dimension reduction is performed using the DDRTree algorithm, and pseudo‐time series analysis of the cells is conducted via the “orderCells()” function to ultimately construct single‐cell developmental trajectories.

State 4 was manually designated as the starting point of the trajectory rather than being automatically determined by Monocle2. This designation was based on the predominant localization of Cluster 3, which showed the highest CytoTRACE2‐derived stemness score in State 4, together with the relatively high CytoTRACE2 scores of cells in this state. The resulting pseudotime was therefore interpreted as an inferred transcriptional ordering rather than a definitive temporal or lineage trajectory.

### 2.8 Cell–Cell Communication

Cell–cell communication was inferred using CellChat (v2.1.2) with the human ligand–receptor interaction database. Two complementary grouping strategies were used. First, epithelial cells were classified as STMN1‐positive malignant epithelial cells (Ep‐STMN1+, Cluster 3), STMN1‐negative malignant epithelial cells (Ep‐STMN1−, Clusters 0, 4, 5, 8, and 9), or non‐malignant epithelial cells (EP‐NM) to characterize communication features associated with the high‐STMN1 malignant epithelial subpopulation. Second, all malignant epithelial cells were independently stratified into STMN1‐high and STMN1‐low groups using the median STMN1 expression level as the cutoff, and CellChat analysis was repeated as a complementary analysis to assess the robustness of the observed communication patterns to an alternative grouping strategy.

For each analysis, CellChat objects were constructed from the gene‐expression matrix and cell annotations. Overexpressed genes and ligand–receptor interactions were identified, and communication probabilities were estimated using computeCommunProb [population.size = FALSE]. Interactions involving fewer than 10 cells were excluded using filterCommunication(min.cells = 10). The resulting CellChat objects were merged for comparative visualization of overall communication networks and specific ligand–receptor interactions using netVisual_circle, netVisual_bubble, and related functions.

### 2.9 Transcription Factor Analysis

To investigate the key upstream regulatory mechanisms of malignant epithelial cells, we performed transcriptional regulatory network analysis of malignant epithelial cells using pySCENIC. We inferred transcription factor (TF) regulatory networks using the GRNBoost2, cisTarget, and AUCell algorithms and calculated the activity values (AUC) of regulons in each cell. Subsequently, based on the pySCENIC results, we extracted the regulatory relationships (regulons) between TFs and target genes. We then used the “get_regulons” function in SCENIC (ver 1.3.1) to obtain a binary TF‐target gene association matrix, where a value of “1” indicates that the transcription factor may regulate the corresponding target gene.

Centered on the target gene, transcription factors with a value of “1” in the corresponding column are screened as potential upstream regulatory factors. Spearman's correlation analysis is then used to assess the correlation between the regulon activity of candidate transcription factors and the expression levels of the target gene. The screening criteria are set as cor.rho > 0.3 and a corrected *p*‐value < 0.05, ultimately identifying significantly correlated key upstream regulatory factors.

### 2.10 Western Blot Assay

Total cellular proteins were extracted and quantified using a BCA Protein Assay Kit (Takara, Japan, Cat. No. T9300A). Equal amounts of protein were separated by SDS‐PAGE and then transferred onto polyvinylidene fluoride (PVDF) membranes. The membranes were incubated overnight at 4°C with the following primary antibodies: rabbit monoclonal anti‐CD44 (CST, USA, Cat. No. 37259, 1:1000), rabbit Polyclonal anti‐Nanog (Proteintech, China, Cat. No. 14295‐1‐AP, 1:1000), rabbit monoclonal anti‐SOX2 (CST, USA, Cat. No. 3579, 1:1000), rabbit monoclonal anti‐OCT4 (CST, USA, Cat. No. 2750, 1:1000), rabbit monoclonal anti‐STMN1 (CST, USA, Cat. No. 13655, 1:1000), and anti‐β‐ACTIN (Proteintech, China, Cat. No. 66009‐1‐Ig, 1:5000). After washing, the membranes were incubated with HRP‐conjugated secondary antibodies at room temperature, including goat anti‐mouse IgG (H + L) (Proteintech, China, Cat. No. SA00001‐1, 1:5000) and goat anti‐rabbit IgG (H + L) (Proteintech, China, Cat. No. SA00001‐2, 1:5000). Protein bands were visualized using an enhanced chemiluminescence (ECL) detection system (Bio‐Rad, USA). All experiments were performed in triplicate.

### 2.11 Tumorsphere Formation Assay

To enrich stem‐like cancer cells, A549 cells (ATCC) were dissociated into single‐cell suspensions and seeded in ultra‐low attachment plates at a density of 1 × 10^3^–5 × 10^3^ cells/mL. Cells were cultured in serum‐free DMEM/F12 medium supplemented with B27 (1×), epidermal growth factor (EGF, 20 ng/mL), and basic fibroblast growth factor (bFGF, 20 ng/mL). The medium was replenished every 2–3 days. After 7–10 days of culture, tumor spheres were counted and imaged under a microscope. Tumor spheres were collected for subsequent RNA and protein extraction to assess the expression of key gene and stemness‐associated markers.

### 2.12 Quantitative Real‐Time PCR (qRT‐PCR)

Total RNA was extracted from A549 cells in the control group and the stemness‐enriched group using the RNAprep Pure Tissue Kit (Tiangen, China; DP431) according to the manufacturer's instructions. The extracted RNA was subsequently reverse‐transcribed into cDNA using the PrimeScript FAST RT Reagent Kit with gDNA Eraser (TaKaRa; RR092A). Quantitative real‐time PCR (qRT‐PCR) was performed using TB Green Premix Ex Taq II (TaKaRa) on a LightCycler 480 II Real‐Time PCR System (Roche, Germany). β‐actin (Sangon Biotech, China) was used as the internal reference gene. The amplification conditions were as follows: 95°C for 5 s and 60°C for 30 s, for a total of 45 cycles. Relative gene expression levels were calculated using the 2^−ΔΔCt^ method. The primer sequences for STMN1, SOX2, NANOG, OCT4, CD44, and the internal reference gene are listed in Table S1.

### 2.13 Statistical Analysis

All statistical analyzes were performed using R software (Version 4.4.1) and GraphPad Prism (Version 9.0). Continuous variables were expressed as mean ± standard deviation (MD ± SD). Comparisons between two groups were conducted using the Student's *t*‐test or Wilcoxon rank‐sum test, while comparisons among multiple groups were performed using one‐way ANOVA or the Kruskal–Wallis test. Correlation analyzes were performed using Pearson or Spearman correlation coefficients. All tests were conducted as two‐sided analyzes, with a significance level of *p* < 0.05.

## 3 Results

### 3.1 Epithelial Cells in Tumor Tissues From Patients With Severe Lung Cancer Exhibit Significant Abnormal Proliferation

First, following standard processing, this study obtained a total of 100,217 cells from samples of normal lung tissue (nLung), early‐stage lung adenocarcinoma (tLung), advanced lung adenocarcinoma (ALC, tL/B), and advanced lung cancer with malignant serous effusions (ALC‐MSE, tL/B_MSE) samples. Using a clustering resolution of 0.7, a total of 27 cell clusters were identified (Figure 1A). Based on known lineage‐specific genes and classic cell marker genes, the 27 cell clusters were annotated as 8 major cell types: epithelial cells (EPCAM, KRT19, KRT18, CDH1), fibroblasts (DCN, THY1, COL1A1, COL1A2), endothelial cells (PECAM1, CLDN5, FLT1, RAMP2), T cells (CD3D, CD3E, CD3G, TRAC), NK cells (NKG7, GNLY, NCAM1, KLRD1), B cells (CD79A, IGHM, IGHG3, IGHA2), myeloid cells (LYZ, MARCO, CD68, FCGR3A), and mast cells (KIT, MS4A2, GATA2) (Figure 1B,D). Analysis of cell proportions revealed that, compared with the normal group, the ratio of epithelial cells to B cells was elevated in the early, advanced, and severe lung cancer groups (tL/B_MSE), while the proportion of myeloid cells was reduced. The proportions of T cells and mast cells were elevated in the early lung cancer group but decreased in the advanced lung cancer and advanced lung cancer with malignant serous effusions (ALC‐MSE) groups (Figure 1C).

**FIGURE 1:**
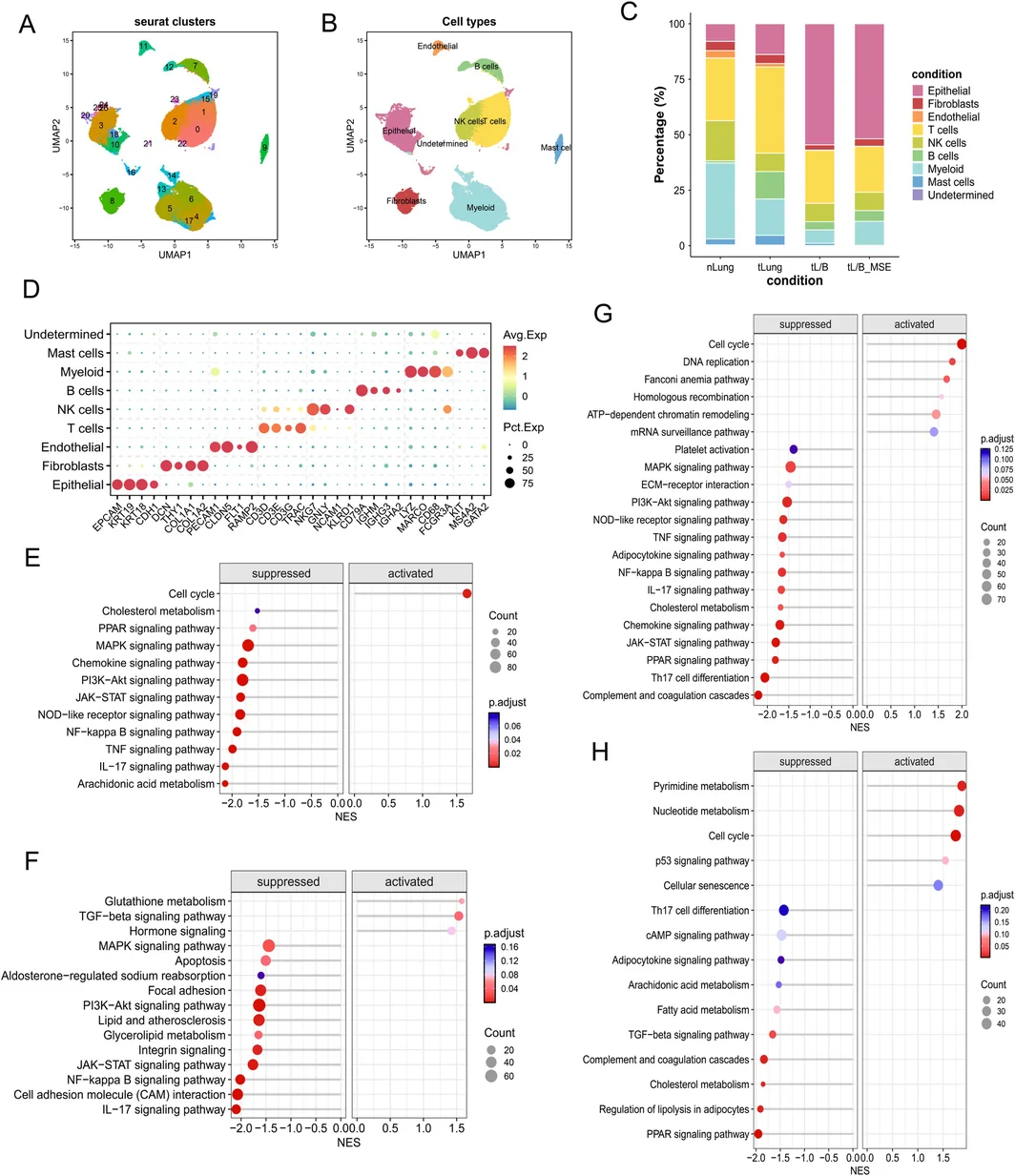
Single‐cell transcriptomic landscape and pathway alterations of epithelial cells across different stages of lung cancer progression. (A) UMAP visualization of all cells, identifying 27 clusters at a clustering resolution of 0.7. (B) Cell‐type annotation based on canonical marker genes, classifying cells into eight major populations: Epithelial cells, fibroblasts, endothelial cells, T cells, NK cells, B cells, myeloid cells, and mast cells. (C) Stacked bar plot showing the cellular composition of each sample group (nLung, tLung, tL/B, and tL/B_MSE). (D) Bubble plot displaying the expression patterns of representative marker genes across major cell types. (E) Gene Set Enrichment Analysis (GSEA) of epithelial cells between the ALC‐MSE and ALC groups. (F) GSEA of epithelial cells between the ALC‐MSE and early‐stage lung cancer groups. (G) GSEA of epithelial cells between the ALC‐MSE and normal lung groups. (H) GSEA of epithelial cells between the advanced lung cancer and normal lung groups.

### 3.2 Stage‐Specific Inflammatory Signaling Changes in Epithelial Cells Across Lung Cancer Progression

Based on single‐cell data showing a significant increase in the proportion of epithelial cells in severe lung cancer with MSE (ALC‐MSE), this study selected epithelial cells as the primary focus of investigation. To investigate functional state changes across different stages of disease progression in advanced lung cancer, Gene Set Enrichment Analysis (GSEA) was performed comparing ALC‐MSE with advanced lung cancer, early‐stage lung cancer, and normal lung tissue, as well as advanced lung cancer versus normal lung tissue.

The results showed that, compared with advanced lung cancer, epithelial cells in ALC‐MSE exhibited significant downregulation of classical inflammatory and immune‐related pathways, including PI3K‐Akt, MAPK, JAK–STAT, NF‐κB, IL‐17, and TGF‐β signaling, along with suppression of cell adhesion, glutathione metabolism, and glycerolipid metabolism pathways (Figure 1E). These findings suggest that, during progression to a more severe disease stage, epithelial cells display a gradual reduction in inflammatory response capacity, intercellular signaling, and antioxidant defense functions, potentially reflecting a state of functional attenuation following sustained inflammatory stimulation.

Compared with early‐stage lung cancer, ALC‐MSE also showed persistent suppression of inflammatory signaling pathways such as PI3K‐Akt, MAPK, JAK–STAT, NF‐κB, and IL‐17, while cell cycle–related programs were significantly upregulated (Figure 1F). These results indicate a progressive decline in inflammatory signaling accompanied by enhanced proliferative activity, suggesting a transition in the tumor microenvironment from an inflammatory state toward immune‐evasive characteristics.

Further comparison with normal lung tissue revealed significant activation of cell cycle, DNA replication, homologous recombination, and Fanconi anemia pathways in ALC‐MSE epithelial cells, whereas inflammatory pathways including MAPK, PI3K‐Akt, NF‐κB, and TNF signaling were markedly downregulated (Figure 1G). This suggests that, at advanced stages, tumor cells gradually acquire a transcriptional state characterized by reduced inflammatory signaling, sustained proliferation, and maintenance of genomic stability.

In addition, compared with normal lung tissue, epithelial cells in advanced lung cancer exhibited activation of cell cycle, nucleotide metabolism, pyrimidine metabolism, p53 signaling, and cellular senescence pathways, while PPAR signaling, cholesterol metabolism, fatty acid metabolism, Th17 cell differentiation, complement and coagulation cascades, and TGF‐β signaling were significantly suppressed (Figure 1H). These findings indicate that lung cancer progression is accompanied by extensive metabolic reprogramming and disruption of inflammatory and immune regulatory networks.

Collectively, across all four comparisons, inflammatory and immune‐related pathways (including NF‐κB, TNF, IL‐17, JAK–STAT, and TGF‐β signaling) showed a consistent decreasing trend in both advanced lung cancer and ALC‐MSE epithelial cells, with further attenuation observed in ALC‐MSE. Meanwhile, lipid metabolism pathways, including cholesterol, fatty acid, and glycerolipid metabolism, were also progressively impaired. Overall, these results indicate that ALC‐MSE is characterized by a coordinated suppression of inflammatory and immune signaling alongside metabolic dysfunction, which may contribute to a biological state favoring sustained proliferation, immune evasion, and malignant progression.

### 3.3 Integrated Identification of Malignant Epithelial Cells and Characterization of Their Inflammatory and Stemness Features

Epithelial‐cell subclustering identified 11 subpopulations (Figure 2A). fastCNV analysis revealed marked heterogeneity in CNV burden among these clusters (Figure 2B). Clusters 3, 5, 9, and 4 showed the highest CNV levels, whereas Cluster 8 exhibited an intermediate level and Cluster 0 showed a relatively lower but still elevated CNV burden compared with the remaining epithelial clusters (Table S2). Clusters 0, 3, 4, 5, 8, and 9 were predominantly derived from tumor samples (Figure S1A). Further validation using epithelial markers (EPCAM, KRT18, KRT19), normal lung epithelial markers (AGER, SFTPC, LAMP3), and tumor‐associated markers (CEACAM5, CEACAM6, MUC1) supported their malignant annotation, including Clusters 0 and 8 despite their relatively lower CNV levels (Figure S1B). Accordingly, these six clusters were classified as malignant epithelial populations (Figure 2C).

**FIGURE 2:**
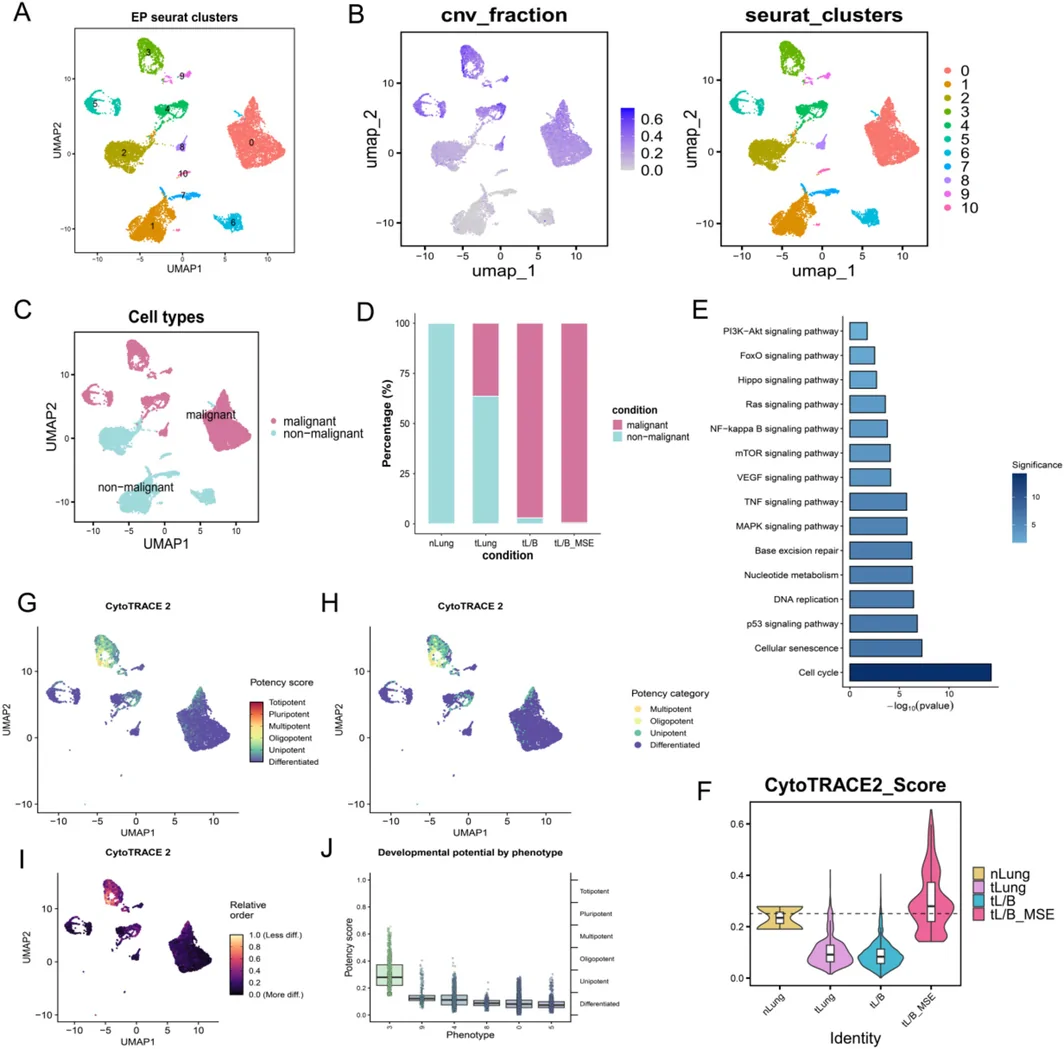
Identification of malignant epithelial cells and characterization of stemness features during lung cancer progression. (A) UMAP visualization of epithelial cell subclusters. (B) Visualization of fastCNV‐derived copy number variation (CNV) fraction scores (left) and the original clustering UMAP (right). (C) Classification of epithelial cells into malignant and non‐malignant populations based on fastCNV‐inferred CNV profiles. (D) Stacked bar plot showing the proportions of malignant and non‐malignant epithelial cells across different sample groups. (E) KEGG pathway enrichment analysis of differentially expressed genes between malignant and non‐malignant epithelial cells. (F) Boxplot showing CytoTRACE2 stemness scores of malignant epithelial cells across the nLung, tLung, tL/B, and tL/B_MSE groups. (G–I) Visualization of CytoTRACE2 stemness scores across malignant epithelial cell subpopulations. (J) Comparison of CytoTRACE2 stemness scores among epithelial cell clusters 0, 3, 4, 5, 8, and 9.

Subsequently, this study further classified the epithelial cells into subpopulations and identified malignant cells. Dimension‐reduction cluster analysis identified a total of 11 epithelial cell subpopulations (Figure 2A). Based on the results of FastCNV analysis, the epithelial cells were further classified into malignant and non‐malignant categories (Figure 2B), with clusters 0, 3, 4, 5, 8, and 9 identified as malignant epithelial cell populations (Figure 2C). Analysis of cellular composition revealed that the proportion of malignant epithelial cells showed a gradual increase in early‐, advanced‐, and severe‐stage lung cancer, reaching the highest proportion in patients with severe lung cancer (Figure 2D).

Based on these findings, a differential functional enrichment analysis was performed on malignant and non‐malignant epithelial cells. The results showed that malignant epithelial cells were significantly enriched in inflammatory and immune‐related signaling pathways such as PI3K‐Akt, MAPK, Ras, NF‐κB, TNF, and IL‐17; this was accompanied by the activation of cell fate regulation pathways such as FoxO, Hippo, and mTOR, as well as a significant enhancement of processes related to proliferation and genomic maintenance, including the cell cycle, DNA replication, the p53 signaling pathway, base excision repair, and nucleotide metabolism (Figure 2E). These findings suggest that malignant epithelial cells exist in a transcriptional state characterized by the synergistic activation of inflammatory response signaling, stress regulatory networks, and proliferation programs. Previous studies have shown that during tumor progression, persistent inflammatory and stress signals (such as the NF‐κB and HIF axes) can promote the acquisition of enhanced self‐renewal capacity and an undifferentiated state in cancer cells by regulating core transcription factors such as Oct4, Sox2, and Nanog, thereby driving the formation and maintenance of tumor stemness (Afify et al. 2022; Jiang et al. 2025; Keith and Simon 2007; Korkaya et al. 2011). Further evaluation of cellular stemness using CytoTRACE2 (v1.1.0) revealed that the overall stemness score was significantly elevated in severe lung cancer tissues (Figure 2F), suggesting that the tumor cell population exhibits a less differentiated state and greater developmental plasticity. Therefore, we hypothesize that malignant epithelial cells in the severe stage of lung cancer may gradually shift toward a less differentiated, stem‐like functional state under prolonged microenvironmental stress.

Therefore, we performed an in‐depth stratification of malignant epithelial cells based on stem cell‐like characteristics to identify key malignant subpopulations closely associated with lung cancer progression. The CytoTRACE2 stem cell‐like score classifies epithelial cell subpopulations into three categories: Multipotent/Oligopotent (high stem cell‐like), Unipotent (moderate), and Differentiated (low stem cell‐like). The results show that most subpopulations of malignant epithelial cells in ALC‐MSE exhibit higher stemness characteristics overall (Figure 2G–I). Among these, Cluster 3 has the highest stemness score and represents the core stemness subpopulation, followed by Clusters 4 and 9, while Clusters 0, 5, and 8 have lower stemness scores (Figure 2J) and may be in a transitional stage of differentiation. Based on this characteristic, we preliminarily identified epithelial cell Clusters 0, 3, 4, 5, 8, and 9 as malignant epithelial subpopulations with core stem cell characteristics in lung cancer.

### 3.4 Trajectory Analysis Identifies Distinct Inflammatory and Stemness‐Associated States in Malignant Epithelial Cells

To elucidate the functional heterogeneity among the aforementioned subpopulations of malignant epithelial cells with varying degrees of stemness, this study further performed KEGG functional enrichment analysis on the six malignant cell clusters (Clusters 0, 3, 4, 5, 8, and 9; Figure 3A). The results showed that Cluster 3 exhibited the strongest stem cell‐like transcriptional program, with significant activation of pathways regulating stem cell pluripotency, Notch, Hippo, TGF‐β, and FoxO, indicating enhanced self‐renewal and stem cell‐like characteristics. At the same time, the pathways of oxidative phosphorylation, the TCA cycle, and mitochondrial autophagy were significantly enriched, suggesting a reliance on mitochondrial metabolism and strong stress adaptability. The remaining subpopulations exhibited distinct functional characteristics: Clusters 4 and 9 were primarily enriched in metabolic reprogramming and energy adaptation pathways; Cluster 5 was enriched in treatment resistance‐related pathways; and Clusters 0 and 8 were enriched in inflammatory activation and immuno‐inflammatory signaling pathways, respectively (Figure 3A). These results reveal significant functional heterogeneity within ALC‐MSE malignant epithelial cells, characterized by high stemness, multifunctional adaptability, and metabolic reprogramming.

**FIGURE 3:**
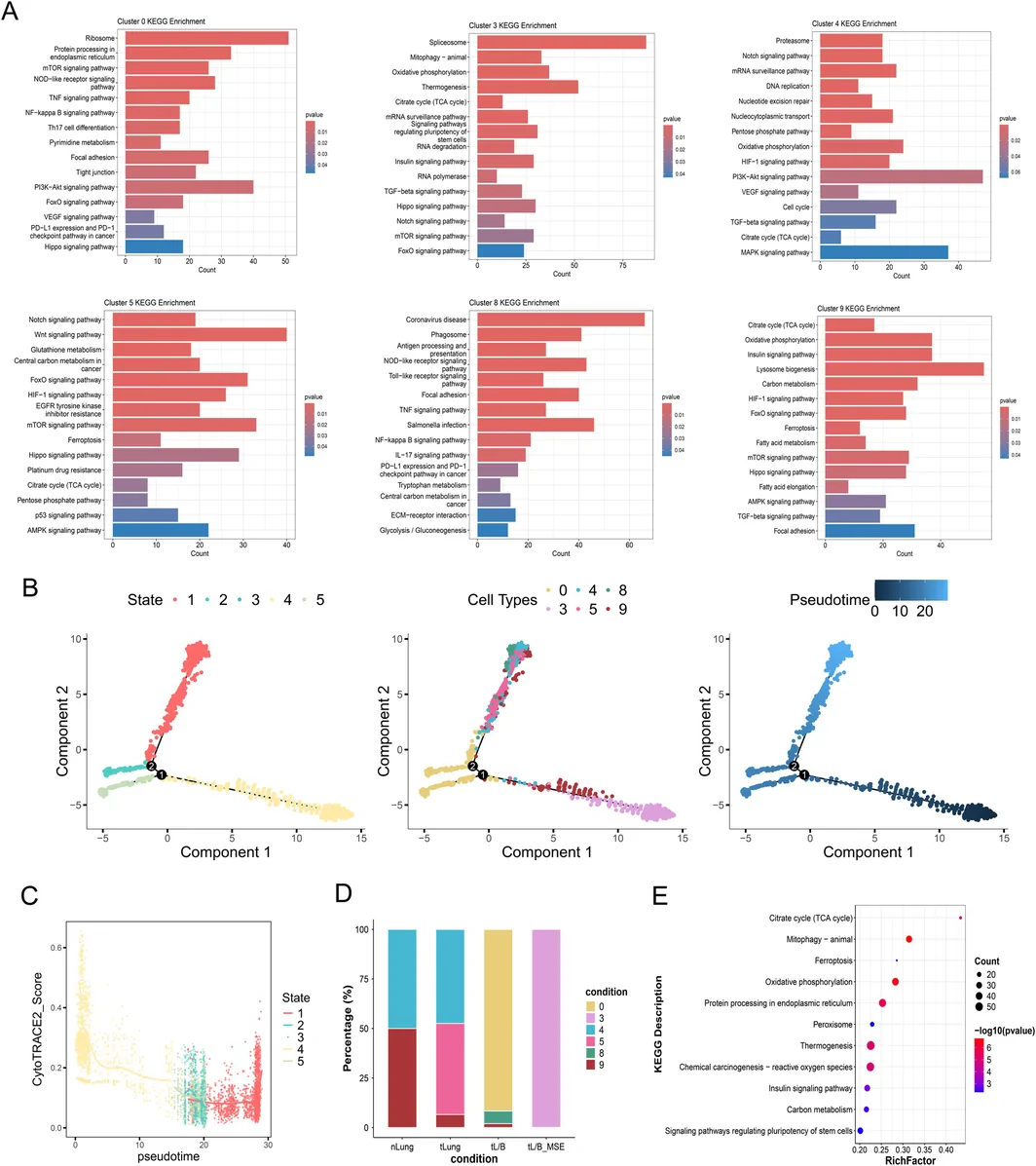
Characterization of malignant epithelial cell heterogeneity and differentiation trajectories. (A) KEGG pathway enrichment analysis revealing functional signatures of six malignant epithelial subclusters (0, 3, 4, 5, 8, and 9). (B) Monocle2‐derived inferred pseudotime trajectory of the six malignant epithelial subclusters, with State 4 manually designated as the starting point. (C) Variation in CytoTRACE2‐based stemness scores along the inferred pseudotime trajectory. (D) Stacked bar plot illustrating the compositional shifts of malignant subpopulations among nLung, tLung, tL/B, and tL/B_MSE groups. (E) Bubble chart summarizing KEGG pathways enriched in State‐4‐specific differentially expressed genes, with bubble size and color representing gene count and significance, respectively.

To further elucidate the evolutionary characteristics of the highly stem‐like subpopulation, this study conducted a pseudo‐temporal analysis of malignant epithelial cells. The results demonstrated that clusters 0 and 8 were predominantly distributed during the intermediate transition phases (state 2 and state 3) and the terminal state (state 1) of the pseudo‐temporal trajectory, whereas the core stem cell Cluster 3 was primarily localized in State 4, representing the initial stage of the trajectory (undifferentiated state) (Figure 5A,B), consistent with the characteristic of impaired differentiation following enhanced tumor cell stemness (Sell and Pierce 1994). Further cell proportion analysis revealed that Cluster 0, which is in an intermediate transitional stage and accompanied by significant activation of immune/inflammatory signaling, accounted for the highest proportion (> 50%) in advanced lung cancer, while Cluster 3, the core stem cell cluster with the highest stemness score, accounted for the highest proportion in severe lung cancer samples (Figure 5C). These findings suggest that inflammation‐associated and high‐stemness malignant epithelial states occupy distinct positions along the inferred transcriptional continuum, with high‐stemness populations being preferentially enriched in severe lung cancer. In this process, inflammation/immune‐related stress states and enhanced stemness exhibit stage‐specific distribution patterns across different cell subpopulations, with a trend toward enrichment of high‐stemness cell populations in severe lung cancer.

Finally, we performed a functional enrichment analysis on the State4‐specific differentially expressed genes primarily distributed in stem‐like subcluster 3. The results showed that State4 is significantly enriched in pathways related to mitochondrial energy metabolism (TCA cycle, oxidative phosphorylation), oxidative stress regulation (ferroptosis), protein processing and homeostasis maintenance (endoplasmic reticulum protein processing, mitochondrial autophagy), and stemness maintenance (stemness regulation, insulin signaling). These results indicate that State4 cells with high stemness not only exhibit undifferentiated characteristics but are also accompanied by significantly enhanced metabolic adaptability and the ability to maintain cellular homeostasis. By remodeling mitochondrial energy metabolism, enhancing stress tolerance, and strengthening protein quality control processes, these cells may gain a stronger survival advantage and greater environmental adaptability, thereby maintaining their stemness and promoting the progression of the disease to a severe stage.

### 3.5 Identification and Independent Validation of STMN1 as a Stemness‐Associated Gene in Malignant Epithelial Cells

Building on the preceding analyzes, we sought to identify genes associated with the high‐stemness malignant epithelial state in ALC‐MSE. Using thresholds of avg_log2FC > 1 and adjusted *p* < 0.05, we identified 1392 genes co‐upregulated in State 4 and ALC‐MSE epithelial cells (Figure 4A). Correlation analysis with CytoTRACE2 scores further prioritized 10 candidate genes, eight of which were evaluable in the TCGA‐LUAD cohort. Among these candidates, STMN1 showed the strongest positive association with the mRNA‐based stemness index (mRNAsi) and was preferentially expressed in high‐stemness Cluster 3 and State 4 (Figure 4B–E), supporting its association with a stem‐like malignant epithelial state.

**FIGURE 4:**
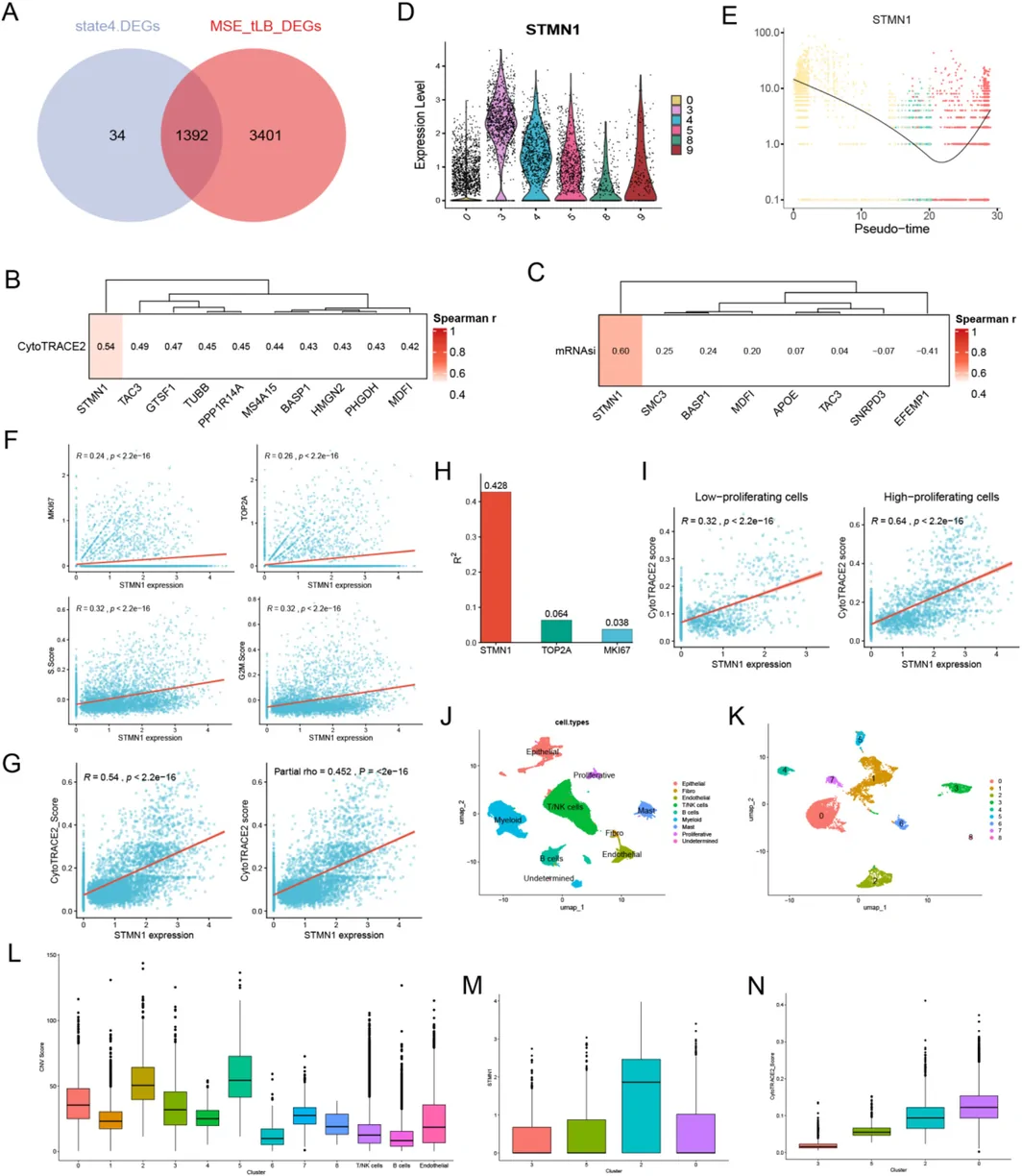
Identification and independent validation of STMN1 as a stemness‐associated gene in malignant epithelial cells. (A) Overlap between genes upregulated in State 4 and those upregulated in ALC‐MSE epithelial cells, highlighting shared candidate genes. (B) Spearman correlations between the top 10 candidate genes and CytoTRACE2‐derived stemness scores. (C) Associations between eight candidate genes available in the TCGA‐LUAD cohort and the mRNA‐based stemness index (mRNAsi). (D) Violin plots showing STMN1 expression across the six malignant epithelial subclusters (0, 3, 4, 5, 8, and 9). (E) Projection of STMN1 expression along the pseudotime trajectory, showing its dynamic expression pattern during differentiation. (F) Spearman correlations between STMN1 expression and the proliferation‐related genes MKI67 and TOP2A, as well as S.Score and G2M.Score. (G) Correlation between STMN1 expression and CytoTRACE2 score before and after adjustment for cell‐cycle scores. (H) Comparison of the variance in CytoTRACE2 scores explained by STMN1, TOP2A, and MKI67 using univariate linear regression models. (I) Correlations between STMN1 expression and CytoTRACE2 scores in low‐ and high‐proliferating malignant epithelial cells. (J) UMAP visualization and major cell‐type annotation of the independent GSE189357 cohort. (K) UMAP visualization of epithelial cell subclusters in GSE189357. (L) inferCNV‐based identification of putative malignant epithelial subclusters in GSE189357. (M) STMN1 expression across malignant epithelial subclusters in GSE189357. (N) CytoTRACE2‐derived stemness scores across malignant epithelial subclusters in GSE189357.

Because malignant epithelial cells also showed enrichment of cell‐proliferation and DNA‐replication programs (Figure 2E), we examined whether the association between STMN1 and stemness could be explained by proliferative activity. STMN1 expression was positively correlated with MKI67, TOP2A, S.Score, and G2M.Score (Figure 4F). After adjustment for cell‐cycle scores, the correlation between STMN1 and CytoTRACE2 remained significant, although the coefficient decreased from 0.54 to 0.452 (Figure 4G). In univariate regression models, STMN1 explained a substantially greater proportion of the variance in CytoTRACE2 scores (*R* ^2^ = 0.428) than TOP2A (*R* ^2^ = 0.064) or MKI67 (*R* ^2^ = 0.038; Figure 4H). Moreover, the STMN1–CytoTRACE2 association remained significant in low‐proliferating malignant epithelial cells (*R* = 0.32) and was stronger in highly proliferating cells (*R* = 0.64; Figure 4I). These findings indicate that the association between STMN1 and stemness is partly related to proliferative activity but is not fully explained by cell‐cycle status, suggesting that STMN1 marks a malignant epithelial state characterized by both enhanced stemness and proliferation.

To independently validate this association, we analyzed an external LUAD single‐cell dataset (GSE189357). After cell‐type annotation and epithelial‐cell subclustering, inferCNV identified clusters 0, 2, 3, and 5 as putative malignant epithelial populations (Figure 4J–L). CytoTRACE2 analysis revealed substantial stemness heterogeneity among these populations. Notably, Cluster 2, which showed the highest STMN1 expression, also exhibited relatively high CytoTRACE2 scores (Figure 4M,N), providing independent support for the association between STMN1 expression and a stem‐like malignant epithelial state. Together, these findings supported the prioritization of STMN1 for subsequent mechanistic and intercellular communication analyzes.

### 3.6 STMN1‐High Malignant Epithelial Cells Exhibit Remodeling of Stromal and Immune Communication

To characterize the microenvironmental context of the STMN1‐associated malignant epithelial state, epithelial cells were classified as STMN1^+^ malignant epithelium (Ep‐STMN1^+^; Cluster 3), STMN1^−^ malignant epithelium (Ep‐STMN1^−^; Clusters 0, 4, 5, 8, and 9), or non‐malignant epithelium (EP‐NM) for CellChat analysis. Fibroblasts represented the dominant communication partners across all epithelial populations, although overall interaction strength was lower in Ep‐STMN1^+^ cells than in Ep‐STMN1^−^ and EP‐NM cells (Figure 5A).

**FIGURE 5:**
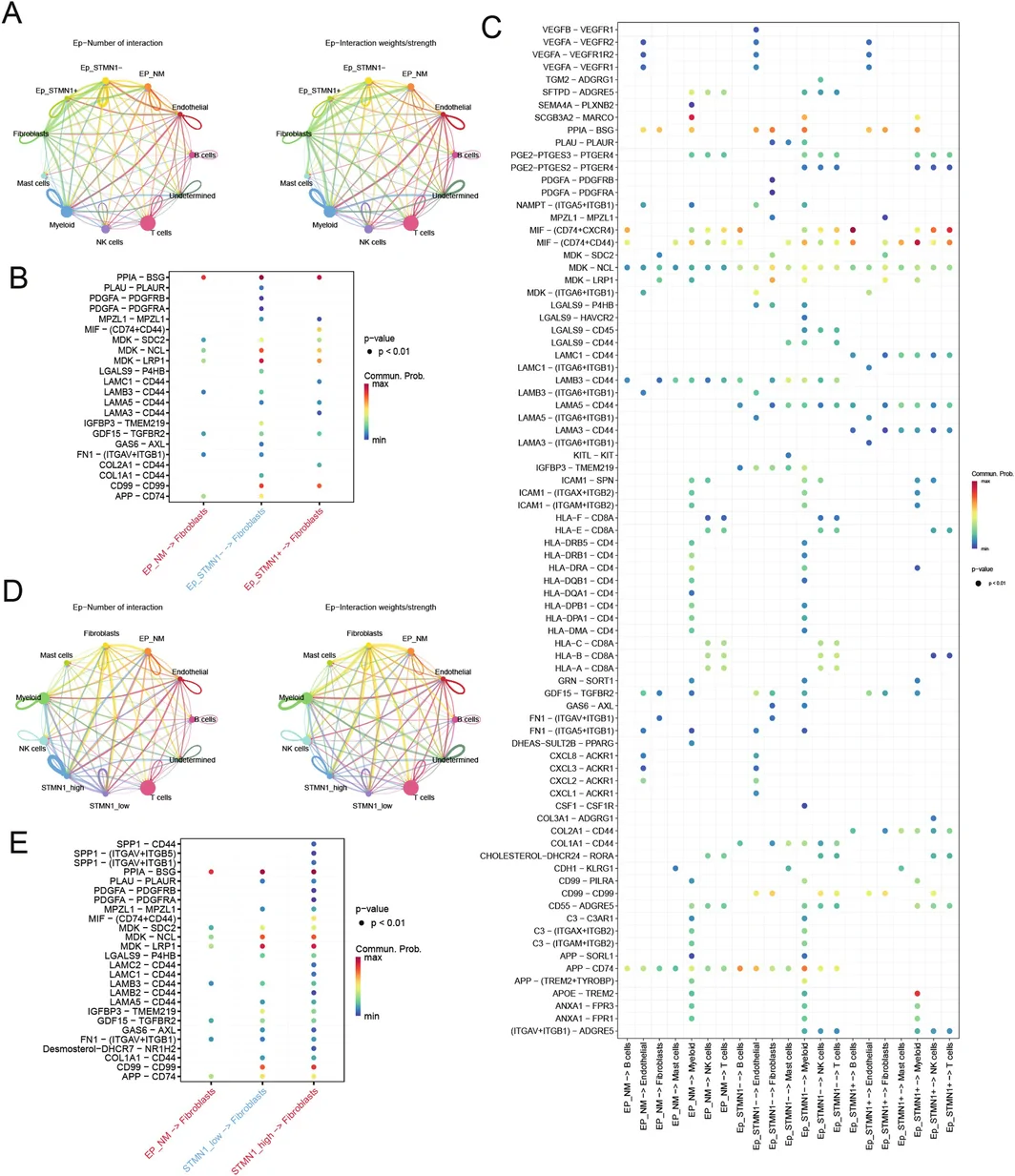
Cell–cell communication landscape of epithelial subpopulations within the tumor microenvironment. (A) Global cell–cell communication network illustrating inferred interactions among all cell types. (B) Ligand–receptor communication between epithelial subpopulations and fibroblasts. (C) Ligand–receptor interactions between epithelial subpopulations and other major cell types. (D) Global cell–cell communication networks comparing the number and strength of inferred interactions involving STMN1‐high and STMN1‐low malignant epithelial cells. (E) Ligand–receptor interactions between STMN1‐high or STMN1‐low malignant epithelial cells and fibroblasts, highlighting differential communication patterns.

Ligand–receptor analysis further revealed distinct fibroblast–epithelial communication programs. In Ep‐STMN1^+^ cells, ECM‐remodeling and adhesion‐associated interactions, including PLAU–PLAUR, PDGFA–PDGFRB, GAS6–AXL, FN1–ITGAV/ITGB1, and COL1A1–CD44, together with stress‐related interactions such as LGALS9–P4HB, IGFBP3–TMEM219, and APP–CD74, were attenuated. In contrast, stemness‐associated interactions, including MIF–CD74/CD44, LAMA3/LAMC1–CD44, and COL2A1–CD44, were enhanced (Figure 5B). These findings indicate a shift from ECM‐ and adhesion‐dominated communication toward signaling potentially supportive of stem‐like states.

Ep‐STMN1^+^ cells also showed reduced immune‐associated communication. Complement‐, myeloid sensing‐, and antigen presentation‐related interactions, including C3–C3AR1, C3–(ITGAX+ITGB2), C3–(ITGAM+ITGB2), APOE–TREM2, ANXA1–FPR1/FPR3, and HLA‐II‐related interactions, were predominantly detected in Ep‐STMN1^−^ or EP‐NM communication networks but were markedly reduced or absent in Ep‐STMN1+ cells (Figure 5C). Collectively, these results identify STMN1^+^ malignant epithelial cells as a distinct communication state characterized by reduced stromal/immune engagement and enhanced stemness‐associated signaling.

In addition, to assess whether these communication patterns depended on the cluster‐based definition of STMN1^+^ cells, all malignant epithelial cells were independently stratified into STMN1‐high and STMN1‐low groups using the median STMN1 expression as the cutoff, and CellChat analysis was repeated. The major communication features were retained, including enhanced MIF–CD74/CD44 signaling and attenuated selected FN1–ITGAV/ITGB1‐associated interactions in STMN1‐high cells (Figure 5D,E). These findings suggest that the observed communication patterns were not solely attributable to the intrinsic characteristics of Cluster 3 but were also associated with STMN1 expression levels.

### 3.7 Prediction of Upstream Transcription Factors That Can Targetly Regulate STMN1 Activity

To identify the upstream transcription factors regulating STMN1 expression, this study screened for transcription factors with potential regulatory relationships with STMN1 based on a TF‐target gene association matrix. Using a column value of “1” for STMN1 in the TF‐target gene matrix as the screening criterion, a total of 8 potential transcription factors were identified. Further analysis using Spearman's correlation coefficient (cor.rho > 0.3, adjusted *p* < 0.05) ultimately identified five key transcription factors: NELFE, MYBL2, FOXN3, POLR2A, and RAD21 (Figure 6A,B). A comparison of the expression patterns of STMN1 and these five upstream transcription factors across different subpopulations of malignant epithelial cells revealed that the expression patterns of NELFE, POLR2A, and RAD21 were highly consistent with that of STMN1, all showing the highest expression levels in core stem‐like Cluster 3 of the malignant epithelial cell subpopulations (Figure 6C). Furthermore, at the whole‐transcriptome level, a trend consistent with STMN1 expression was observed, with all three genes showing significantly higher expression in patients with advanced lung cancer accompanied by MSE (ALC‐MSE) (Figure 6D; *p* < 0.05). These results suggest that NELFE, POLR2A, and RAD21 may be key upstream transcription factors regulating the high expression of STMN1 in the stem‐like core Cluster 3.

**FIGURE 6:**
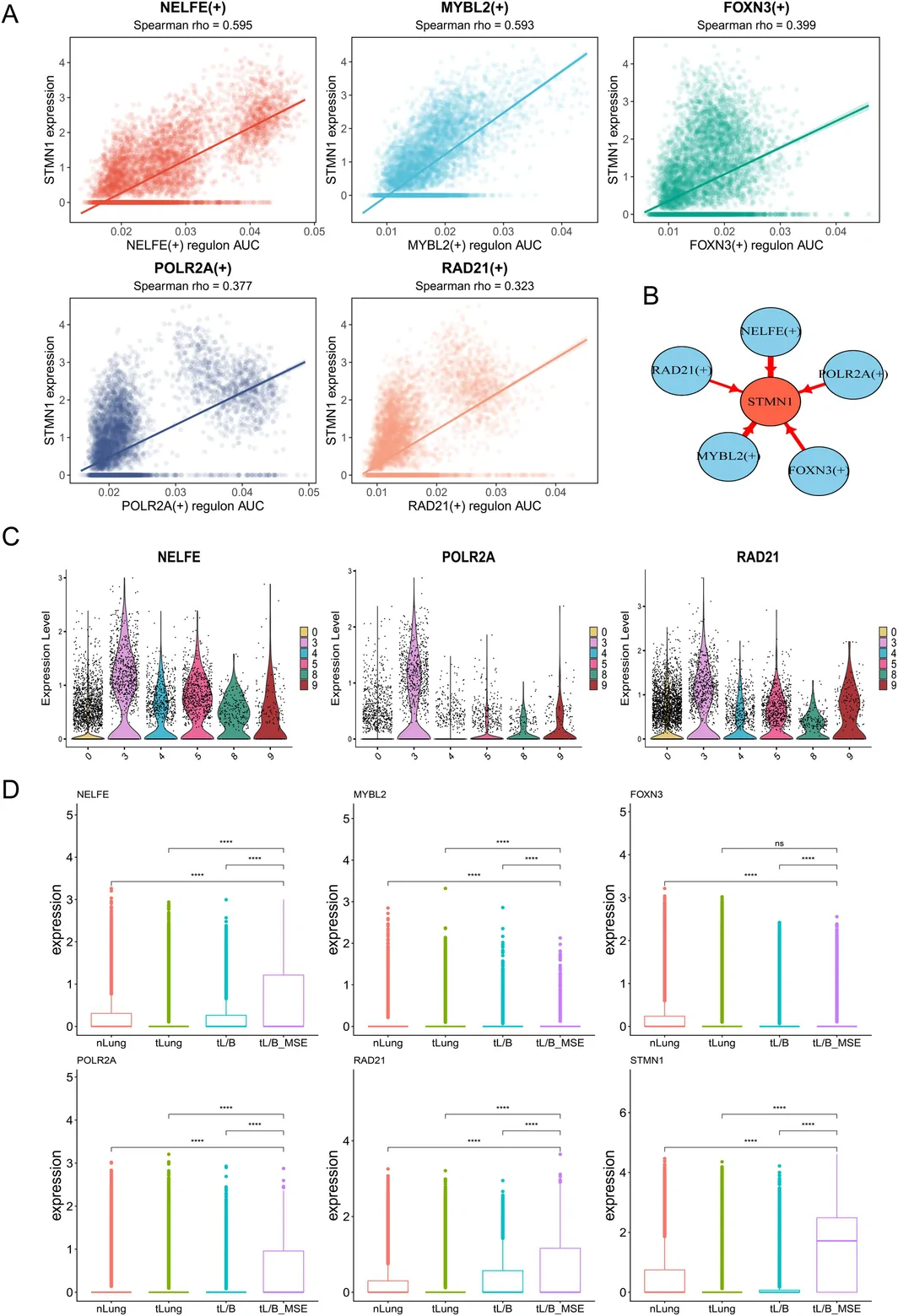
Screening and regulatory network of upstream transcription factors regulating STMN1. (A) Spearman correlation scatter plot between upstream transcription factors and STMN1 gene expression. (B) Schematic diagram of the regulatory network between upstream transcription factors and the STMN1 gene. (C) Expression profiles of upstream transcription factors in malignant epithelial cell subpopulations. (D) Box plot showing expression levels of STMN1 and its upstream transcription factors across different cell populations.

### 3.8 STMN1 Expression Is Elevated in Stemness‐Enriched A549 Cells

To investigate whether STMN1 participates in the formation and maintenance of the stemss phenotype in lung adenocarcinoma cells, this study employed tumor spheroid formation assays to enrich A549 cells with stem‐like‐induced characteristics, followed by proteomic and transcriptomic analyzes. Western blot and qRT‐PCR results demonstrated that compared to the control group, both STMN1 protein expression and mRNA levels were significantly elevated in stem‐like‐induced A549 cells (Figure 7A,B; *p* < 0.05). Additionally, the tumor spheroid formation capacity was markedly enhanced in the stem‐like‐induced group, evidenced by a significant increase in the number of tumor spheroids (Figure 7C; *p* < 0.05).

**FIGURE 7:**
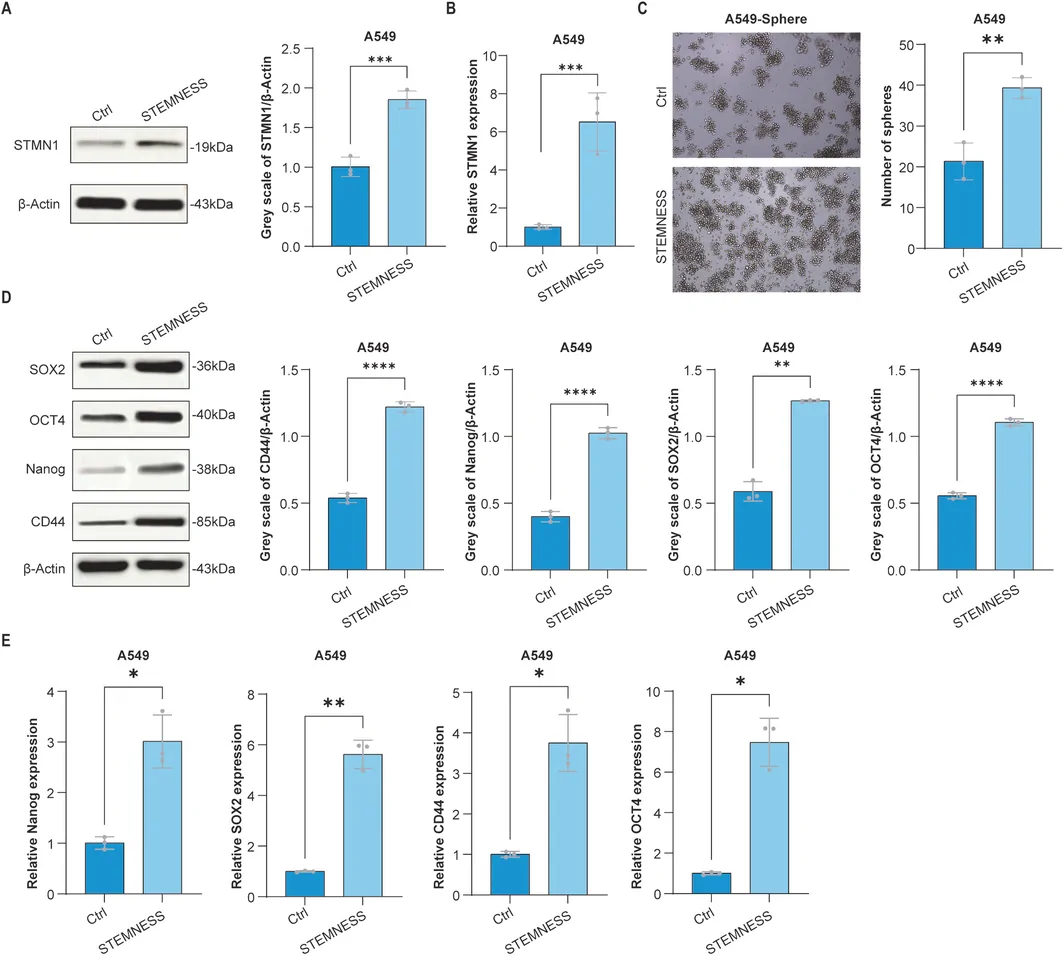
Validation of STMN1 expression and stemness characteristics in stemness‐enriched A549 cells. (A) Representative Western blot images and corresponding quantification of STMN1 protein expression in stemness‐enriched A549 cells and control A549 cells, based on three independent Western blot experiments. (B) Relative mRNA expression of STMN1 in stemness‐enriched and control A549 cells determined by quantitative real‐time PCR (qRT‐PCR). (C) Representative images and quantitative analysis of tumor sphere formation in stemness‐enriched and control A549 cells. (D) Western blot analysis and corresponding quantification of stemness‐associated markers (SOX2, NANOG, OCT4, and CD44) in stemness‐enriched and control A549 cells. (E) Relative mRNA expression levels of SOX2, NANOG, OCT4, and CD44 in stemness‐enriched and control A549 cells determined by qRT‐PCR.

To further validate the stemness enrichment effect, we examined the expression of several classic stemness markers. The results showed that the protein and mRNA expression levels of SOX2, NANOG, OCT4, and CD44 in A549 cells after stemness induction were significantly higher than those in the control group (Figure 7D,E, *p* < 0.05). These findings indicate that STMN1 expression levels are significantly positively correlated with the stemness state of lung adenocarcinoma cells, suggesting that it may be involved in the maintenance of stemness in these cells.

### 3.9 NELFE–STMN1 Axis Promotes Stemness‐Associated Phenotypes in Lung Cancer Cells

To investigate the role of STMN1 in lung cancer cell stemness, STMN1 knockdown and overexpression models were established in A549 and PC‐9 cells. Western blotting confirmed efficient STMN1 knockdown and overexpression (Figure 8A,B).

**FIGURE 8:**
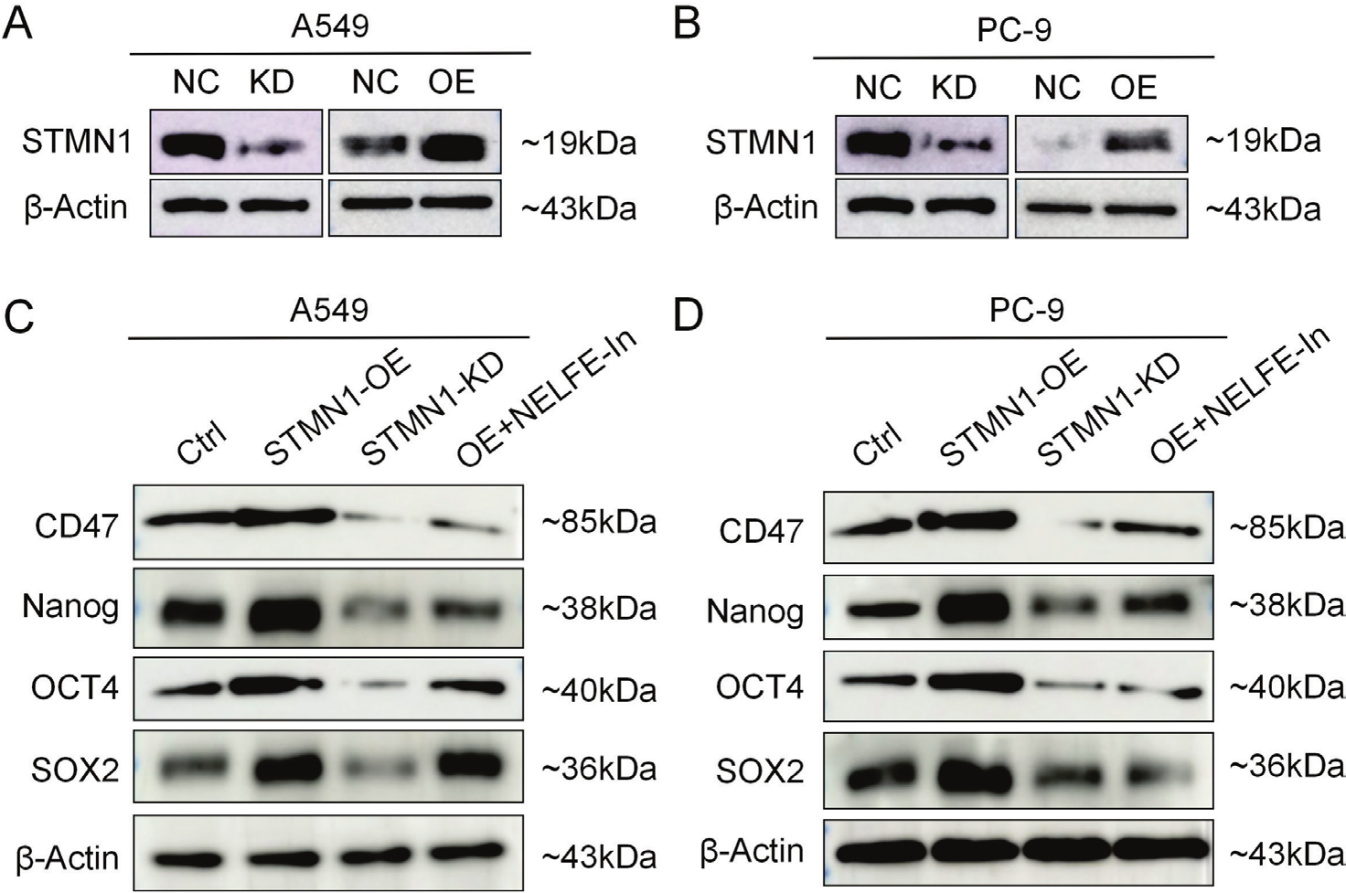
NELFE inhibition attenuates STMN1‐mediated expression of stemness‐associated proteins in lung cancer cells. (A, B) Western blot analysis confirming STMN1 knockdown (KD) and overexpression (OE) efficiency in A549 (C) and PC‐9 (D) cells. (C, D) Western blot analysis of the stemness‐associated proteins CD47, Nanog, OCT4, and SOX2 in A549 (C) and PC‐9 (D) cells subjected to Ctrl, STMN1‐OE, STMN1‐KD, or STMN1‐OE plus NELFE inhibitor (NELFE‐In) treatment. β‐Actin was used as the loading control.

STMN1 overexpression increased the expression of the stemness‐associated proteins CD47, Nanog, OCT4, and SOX2, whereas STMN1 knockdown reduced their expression in both A549 and PC‐9 cells (Figure 8C,D), indicating that STMN1 positively regulates stemness‐associated phenotypes. Furthermore, treatment with the NELFE inhibitor NELFE‐In in STMN1‐overexpressing cells attenuated the upregulation of CD47, Nanog, OCT4, and SOX2 (Figure 8C,D). These findings suggest that NELFE contributes to STMN1‐mediated maintenance of lung cancer cell stemness and support a regulatory role for the NELFE–STMN1 axis.

## 4 Discussion

Advanced lung cancer with malignant serous effusion (ALC‐MSE) represents a critical clinical manifestation of disease progression to an advanced stage, characterized by increased tumor burden, diminished treatment response, and poor prognosis. However, systematic understanding of the functional status and regulatory mechanisms of malignant cells in ALC‐MSE remains limited. This study, utilizing single‐cell transcriptomics, multidimensional stemness assessment, and cell communication analysis, identified a population of highly stem‐like malignant epithelial cells in ALC‐MSE that exhibit high STMN1 expression. The findings suggest that inflammatory signaling dysregulation, enhanced stemness, and remodeling of the microenvironmental communication network may collectively contribute to the pathogenesis and progression of advanced lung cancer (ALC‐MSE).

First, our study found that as the disease progresses from early‐stage lung cancer to advanced‐stage lung cancer and the ALC‐MSE stage, the proportion of malignant epithelial cells gradually increases, while the overall stemness score continues to rise. GSEA analysis further revealed that inflammatory and immune regulatory pathways (such as NF‐κB, TNF, IL‐17, JAK–STAT, and TGF‐β pathways) exhibited a sustained downregulation trend in ALC‐MSE, whereas processes related to the cell cycle, DNA replication, and DNA damage repair were significantly activated. This phenomenon suggests that advanced lung cancer does not manifest as persistently heightened inflammation, but rather is more likely to represent a distinct biological state characterized by attenuated inflammatory responses, imbalanced immune regulation, and sustained proliferative advantage. Recent studies have shown that prolonged inflammatory stimulation and hypoxic stress can induce phenotypic remodeling in tumor cells and promote the formation and maintenance of cancer stem cells (CSCs) (Shang et al. 2025; Taniguchi and Karin 2018). Based on these findings, we hypothesize that the persistent microenvironmental stress in ALC‐MSE may drive certain malignant cells to gradually transition toward a low‐differentiated, highly plastic stem‐like state.

Further CytoTRACE (Ge et al. 2025; Kang et al. 2025) and pseudo‐time‐series analyzes confirmed the presence of significant stemness heterogeneity within malignant epithelial cells. Among these, Cluster 3 exhibited the highest stemness score and was primarily distributed in State 4 at the beginning of the pseudo‐time‐series trajectory, whereas Cluster 0, which is enriched in advanced lung cancer, was mainly located in the transitional stage characterized by active inflammatory signaling. Notably, as the disease progresses, the cell population exhibits a trend toward enrichment from an inflammation‐active state to a highly stem‐like, undifferentiated state. State 4‐specific genes are further enriched in pathways such as oxidative phosphorylation, the TCA cycle, mitochondrial autophagy, endoplasmic reticulum protein processing, and ferroptosis, suggesting that highly stem‐like cells not only exhibit undifferentiated characteristics but are also accompanied by enhanced metabolic adaptation and stress tolerance. A growing body of evidence indicates that cancer stem cells can maintain energy supply and cellular homeostasis through metabolic reprogramming, thereby gaining a sustained survival advantage in adverse microenvironments (Farah et al. 2025; Lee et al. 2025; Tan et al. 2025; Tylichova et al. 2026). The findings of our study are highly consistent with the aforementioned perspectives.

This study focuses on key molecules involved in maintaining cellular stemness. Through multidimensional screening, we identified STMN1 as a core factor regulating high stemness in ALC‐MSE cells, which is consistently highly expressed in epithelial cell core stemness subpopulation 3 and State 4. STMN1 encodes microtubule destabilizing protein (Liu et al. 2023), which has been shown to be involved in cell cycle regulation, proliferation, and metastasis in various tumors (Hsieh et al. 2010; Kuang et al. 2016; Zeng et al. 2024). Its high expression has been linked to poor prognosis in lung cancer patients (Nie et al. 2015). However, its role in maintaining stemness in advanced lung cancer remains poorly understood. In this study, in vitro tumor spheroid induction experiments further confirmed that enhanced stemness is accompanied by a significant upregulation of STMN1 expression, suggesting that STMN1 may be involved in maintaining the stemness phenotype of lung adenocarcinoma cells. Combined with SCENIC analysis results, transcriptional regulatory factors such as NELFE, POLR2A, and RAD21 may collectively participate in the regulation of STMN1‐related transcriptional programs, suggesting that highly stem‐like ALC‐MSE cells may rely on a multilevel transcriptional regulatory network to maintain their malignant state.

Furthermore, this study also revealed significant communication remodeling between STMN1‐high‐expressing cells and the tumor microenvironment. Compared with other epithelial cells, STMN1^+^ malignant epithelial cells exhibited markedly weakened ECM remodeling and adhesion‐related signaling with fibroblasts, while stemness‐related signaling, such as MIF‐CD74/CD44, was significantly enhanced. Combined with existing research, this indicates that the MIF‐CD44/CD74 signaling axis plays a key role in bridging microenvironmental stress signals and the intrinsic stemness program of tumor stem cells in various tumors (Du et al. 2013; He et al. 2026). These results further support the notion that STMN1^+^ malignant epithelial cells exhibit stronger stemness maintenance and niche‐dependent characteristics. At the same time, we also observed that multiple ligand–receptor axes associated with complement activation, antigen presentation, and myeloid cell immune recognition (such as C3, ANXA1, and MHC‐II‐related molecules) were extensively absent or significantly attenuated in STMN1^+^ cells. Previous studies have suggested that these molecules are typically closely associated with persistent inflammatory stimulation and the activation of immune surveillance (Axelrod et al. 2019; Chaumonnot et al. 2021; Zheng et al. 2024); their overall signaling silencing in STMN1^+^ malignant epithelial cells suggests that this cell subset may evade immune recognition and adapt to tumor progression by restructuring immune communication networks. Combined with the results of pseudo‐temporal analysis, it was further found that STMN1^−^epithelial cells, including those in Clusters 0 and 8, were primarily enriched in intermediate stages of differentiation and exhibited strong inflammatory responses and immune interaction characteristics; however, as cells evolved toward a terminal malignant state, inflammation‐related signaling gradually weakened, and stem cell‐related signaling gradually became dominant. These findings collectively suggest that STMN1‐positive malignant epithelial cells may represent a transitional endpoint characterized by “low immune interaction–high stem cell maintenance”: on one hand, they reduce interaction intensity with the microenvironment and immune system by attenuating signals related to ECM adhesion and antigen presentation; on the other hand, they enhance stem cell‐supporting pathways such as the MIF axis, thereby reshaping tumor niche dependence and achieving functional evolution from an inflammatory response‐oriented epithelial state to a stem cell‐dominated malignant state.

In summary, this study reveals the existence of a population of highly stem‐like malignant epithelial cells in ALC‐MSE characterized by high STMN1 expression, the formation of which may be accompanied by suppression of inflammatory signaling, enhanced metabolic adaptation, and remodeling of microenvironmental communication. STMN1 is not only an important biomarker of the stemness state in advanced lung cancer but may also serve as a key regulatory node linking tumor stemness and microenvironmental remodeling, providing a new theoretical basis for understanding the molecular mechanisms of ALC‐MSE and developing stemness‐targeted therapeutic strategies.

However, this study still has certain limitations. First, the single‐cell analyzes were mainly based on publicly available datasets, and the number of ALC‐MSE samples was limited; therefore, validation in larger independent clinical cohorts is warranted. Second, although STMN1 knockdown and overexpression in A549 and PC‐9 cells consistently altered the expression of core stemness‐associated markers, direct functional validation using tumorsphere formation assays after STMN1 manipulation remains lacking. Thus, the current findings support the involvement of STMN1 in stemness‐associated phenotypes but do not yet definitively establish its role in maintaining self‐renewal capacity. Third, the predicted cell–cell communication network, particularly the MIF–CD74/CD44 axis, is based on computational inference. Although this interaction was consistently identified using two independent STMN1‐based stratification strategies, its functional contribution remains to be established through ligand/receptor blockade, conditioned‐medium or co‐culture experiments, spatial validation, and in vivo studies.

## Author Contributions

**Yanmei Chen:** conceptualization, writing – original draft, visualization, validation, formal analysis, resources. **Sisi Chen:** visualization, formal analysis, project administration, data curation. **Biao Cheng:** investigation, validation, visualization, formal analysis, software, supervision. **Yuzhu Tang:** conceptualization, methodology, software, visualization. **Yiyan Huang:** validation, project administration, formal analysis. **Miao Chen:** writing – review and editing, visualization, software, data curation, resources, supervision, funding acquisition.

## Funding

The authors have nothing to report.

## Conflicts of Interest

The authors declare no conflicts of interest.

## Supporting information


**Figure S1:** Distribution and marker expression profiles of epithelial cell subclusters. (A) Relative proportions of epithelial cell subclusters (clusters 0–10) across nLung, tLung, tL/B, and tL/B_MSE samples. (B) Violin plots showing the expression of epithelial markers (EPCAM, KRT18, and KRT19), normal lung epithelial‐associated markers (AGER, SFTPC, and LAMP3), and tumor‐associated markers (CEACAM5, CEACAM6, and MUC1) across epithelial cell subclusters.
**Table S1:** The qRT‐PCR primer sequences are as follows.
**Table S2:** Summary of fastCNV‐derived CNV metrics across epithelial cell subclusters.

## Data Availability

The data that support the findings of this study are available on request from the corresponding author. The data are not publicly available due to privacy or ethical restrictions.
