# Supplementary material for: Single‐Cell Dissection of Inflammatory Suppression and Stemness Plasticity Reveals STMN1‐Associated Malignant Epithelial Cells in Severe Lung Cancer
Source: Chem Biol Drug Des. 2026 Oct 21;108(4):e70420. doi: 10.1111/cbdd.70420 (PMC13685055; doi:10.1111/cbdd.70420)
Supplement: Supplementary file 1 [file CBDD-108-e70420-s001.docx]

**Supplementary Figure S1. Distribution and marker expression profiles of epithelial cell subclusters.**

(A) Relative proportions of epithelial cell subclusters (clusters 0–10) across nLung, tLung, tL/B, and tL/B_MSE samples. (B) Violin plots showing the expression of epithelial markers (EPCAM, KRT18, and KRT19), normal lung epithelial-associated markers (AGER, SFTPC, and LAMP3), and tumor-associated markers (CEACAM5, CEACAM6, and MUC1) across epithelial cell subclusters.

**Supplementary Table S1. The qRT-PCR primer sequences are as follows**

| Gene | Forward primer (5′–3′) | Reverse primer (5′–3′) |
| --- | --- | --- |
| STMN1 | TCAGCCCTCGGTCAAAAGAAT | TTCTCGTGCTCTCGTTTCTCA |
| SOX2 | GCCGAGTGGAAACTTTTGTCG | GGCAGCGTGTACTTATCCTTCT |
| NANOG | TTTGTGGGCCTGAAGAAAACT | AGGGCTGTCCTGAATAAGCAG |
| OCT4 | CTGGGTTGATCCTCGGACCT | CCATCGGAGTTGCTCTCCA |
| CD44 | CTGCCGCTTTGCAGGTGTA | CATTGTGGGCAAGGTGCTATT |
| β-actin | CATGTACGTTGCTATCCAGGC | CTCCTTAATGTCACGCACGAT |

**Supplementary Table S2. Summary of fastCNV-derived CNV metrics across epithelial cell subclusters**

| seurat_clusters | n_cells | mean_cnv | median_cnv | high_cnv_prop |
| --- | --- | --- | --- | --- |
| 3 | 1078 | 0.40 | 0.3716 | 0.8145 |
| 5 | 931 | 0.40 | 0.3945 | 0.8625 |
| 9 | 230 | 0.37 | 0.3532 | 0.5957 |
| 4 | 961 | 0.33 | 0.3073 | 0.5338 |
| 8 | 306 | 0.30 | 0.2729 | 0.4118 |
| 0 | 4393 | 0.25 | 0.2431 | 0.1703 |
| 2 | 2376 | 0.13 | 0.1284 | 0.0055 |
| 10 | 185 | 0.07 | 0.0734 | 0.0000 |
| 6 | 825 | 0.04 | 0.0321 | 0.0073 |
| 1 | 2887 | 0.03 | 0.0092 | 0.0007 |
| 7 | 430 | 0.02 | 0.0138 | 0.0000 |
